# The Role of Chemopreventive Agents in the Management of Oral Potentially Malignant Disorders and Oral Cancer: A Narrative Review

**DOI:** 10.1002/cnr2.70559

**Published:** 2026-04-24

**Authors:** Lana Sayal, Omar hamadah, Eyad Chatty, Anas Abdo, Sana Aghbari, Ali Munasser, Amirah Alnour

**Affiliations:** ^1^ Faculty of Dentistry, Oral Medicine Department Alwatanyia Private University Hama Syria; ^2^ University of Aden Aden Yemen; ^3^ Faculty of Dentistry, Oral Medicine Department Damascus University Damascus Syria; ^4^ Faculty of Medicine, Pathology Department Damascus University Damascus Syria; ^5^ Faculty of Dentistry, Endodontic Department Damascus Syria; ^6^ International University of Science and Technology Daraa Syria; ^7^ Faculty of Dentistry, Oral Medicine Department University of Aden Aden Yemen; ^8^ Faculty of Dentistry, Oral and Maxillofacial Surgery Department University of Aden Aden Yemen; ^9^ Faculty of Dentistry, Oral and Maxillofacial Pathology Department Damascus University Damascus Syria

**Keywords:** bioengineer, chemopreventive agents, oral potentially malignant disorders, oral squamous cell carcinoma, phytochemical oral carcinogenesis

## Abstract

**Background:**

Oral squamous cell carcinoma (OSCC) is a common and aggressive cancer sometimes subsequent to oral potentially malignant disorders (OPMDs). Notwithstanding progress in surgical, radiotherapeutic, and chemotherapeutic interventions, survival rates for OSCC continue to be inadequate and are associated with significant functional impairment, underscoring the necessity for preventive and interceptive therapeutic approaches. Chemoprevention has emerged as a viable strategy to inhibit, postpone, or reverse oral carcinogenesis by targeting the molecular and cellular processes implicated in malignant transformation.

**Recent Findings:**

This review synthesizes current evidence on chemopreventive agents investigated in OPMDs and OSCC. Relevant literature was analyzed focusing on natural compounds, synthetic drugs, and targeted biological therapies, as well as emerging delivery approaches.

Significant emphasis is placed on bioactive phytochemicals such as retinoids, carotenoids (β‐carotene and lycopene), curcumin, resveratrol, black raspberries, and vitamin E, alongside synthetic agents including cyclooxygenase‐2 inhibitors and epidermal growth factor receptor (EGFR)–targeted therapies. These drugs demonstrate chemopreventive effects via modulating oxidative stress, inflammation, cell cycle regulation, apoptosis, angiogenesis, epithelial–mesenchymal transition, and immune evasion in the tumor microenvironment. Clinical and experimental investigations examined in this review reveal inconsistent, although promising results, including the regression of dysplasia, decreased rates of malignant transformation, and enhanced molecular risk profiles in OPMDs. Nonetheless, obstacles persist about long‐term effectiveness, ideal dose, toxicity, and patient adherence.

**Conclusion:**

Chemopreventive medicines, especially when utilised in combination and through sophisticated delivery systems, signify a prospective adjunct or alternative approach in the care of OPMDs and OSCC, with the capacity to diminish disease burden and enhance patient outcomes.

## 1 Introduction

Oral squamous cell carcinoma (OSCC) is the predominant cancer of the oral cavity and constitutes a significant worldwide health challenge, with consistently low survival rates despite advancements in surgical, radiotherapeutic, and chemotherapeutic interventions [1].

Based on the global burden 2020 (Cancer Statistics), oral carcinoma is one of the most prevalent malignancies on a global scale. Globally, the age‐adjusted oral cancer incidence rate was 6.0 per 10 000 in men and 2.3 per 10 000 in women [2]. And it has a poor survival rate at roughly 5 years of fewer than 40%–50%, and disability‐standardized life years have risen by 87.1%. The five‐year survival rate of OSCC seldom surpasses 40%–50%, and therapy often entails considerable functional, esthetic, and psychological morbidity, highlighting the necessity for more efficacious preventive and early intervention measures [3, 4].

New data revealed an increasing increase in the prevalence of oral carcinoma among young persons under 40 years old during the previous 30 years [5]. Historically, the incidence of oral cancer has been greater in men than in women; however, this disparity is diminishing with time. It affects the front two‐thirds of the tongue, buccal mucosa, mucosal surfaces of the lips, floor of the mouth, hard and soft palate, gingiva, retromolar region, and any other region of the oral cavity [6]. A characteristic aspect of OSCC is its frequent antecedence by clinically recognisable oral potentially malignant disorders (OPMDs), including oral leukoplakia, oral erythroplakia, oral submucous fibrosis, oral lichen planus, and oral proliferative verrucous leukoplakia.

These lesions demonstrate varying yet well‐established risks of malignant transformation, with reported rates between 3% and over 30%, contingent upon clinical and histological parameters. Histologically, oral potentially malignant disorders exhibit a spectrum of epithelial changes, ranging from hyperkeratosis and dysplasia to carcinoma in situ, illustrating the multistep process of mouth carcinogenesis. This extended premalignant stage provides a crucial opportunity for therapeutic intervention prior to the onset of invasive cancer [7].

Oral leukoplakia is considered as the most common non‐neoplastic lesion in the oral cavity. It is predominantly white lesion of the oral mucosa of questionable risk, after not included other definable lesions that have no potenial risk for cancer, with a global incidence of 4.11%, malignant transformation (MT) was 3.5%–9.8%, and a varied rates between 0.13% and 40.8% [8, 9, 10].

Other OPMDs that are related to a high risk of MT include proliferative verrucous leukoplakia, erythroplakia, oral submucosal fibrosis (OSMF), oral lichen planus, and palatal lesions from reverse cigar smoking. The reported rates of malignant transition to OSCC from possibly “OPMDs.” range from 3% to 50%, of which oral leukoplakia occupies 17%–35%. Histologically, these lesions typically demonstrate hyperkeratosis and/or hyperplasia, dysplasia (mild, moderate, and severe), and ultimately in situ carcinoma (ISC), with a significant malignant transformation rate of 5%–36% [11]. Traditional therapies for OSCC include chemotherapy, radiation therapy, and surgery. These treatments have associated side effects, such as functional loss, cosmetic modifications, xerostomia, mucositis, dental diseases, hearing abnormalities, and thyroid disorders. Additionally, patients endure chemotherapy‐associated symptoms such as hair loss, nausea, vomiting, and exhaustion, which limit treatment adherence. The present management of OPMDs predominantly involves surgical excision, monitoring, and adjustment of risk factors. Surgical interventions may not consistently avert recurrence or malignant transformation and frequently result in functional impairment, especially when lesions are multifocal or situated in anatomically intricate areas of the oral cavity. Furthermore, traditional OSCC treatments are predominantly reactive, focusing on existing malignancies rather than preventing carcinogenic advancement. These constraints have stimulated increasing interest in chemoprevention as an adjunctive technique to diminish cancer incidence, postpone advancement, or avert recurrence. The term “chemoprevention,” comprising natural and synthetic drugs, is commonly regarded as preventing or suppressing cancer by hindering the initiation of pre‐malignant lesions (blocking agents) or by influencing critical molecules that control cell differentiation, proliferation, and death. Chemoprevention denotes the application of natural, synthetic, or biological agents to impede, reverse, or suppress carcinogenesis by addressing critical molecular events associated with cancer initiation and progression.

## 2 Molecular Pathways Involved in Oral Carcinogenesis and Targets for Chemoprevention

Oral carcinogenesis is a sequential process mediated by the gradual aggregation of genetic and epigenetic alterations affecting several key molecular signaling pathways. Among the most frequently implicated pathways is the epidermal growth factor receptor (EGFR) signaling cascade, which controls cellular proliferation, survival, and differentiation. Overexpression or activation of EGFR has been identified in a significant proportion of oral squamous cell carcinomas and is associated with enhanced tumor growth and resistance to apoptosis.

Another critical pathway involved in oral tumorigenesis is the PI3K/AKT/mTOR signaling pathway, which is crucial for the regulation of cell survival, metabolism, and angiogenesis. Uncontrolled activation of this pathway has been frequently observed in oral potentially malignant disorders (OPMDs) and OSCC and contributes to uncontrolled cellular proliferation and resistance to programmed cell death [12, 13].

Alterations in tumor suppressor genes, particularly mutations in the TP53 gene, also represent a hallmark of oral carcinogenesis. Loss of p53 function disrupts normal cell cycle regulation and DNA repair mechanisms, thereby facilitating the accumulation of genetic damage and malignant transformation.

In addition, inflammatory signaling pathways, including activation of the NF‐κB pathway, have been shown to promote tumor initiation and progression by regulating cytokine production, cell survival, and the tumor microenvironment. Chronic inflammation is increasingly recognized as an important contributor to the progression of OPMDs toward OSCC.

**FIGURE 1:**
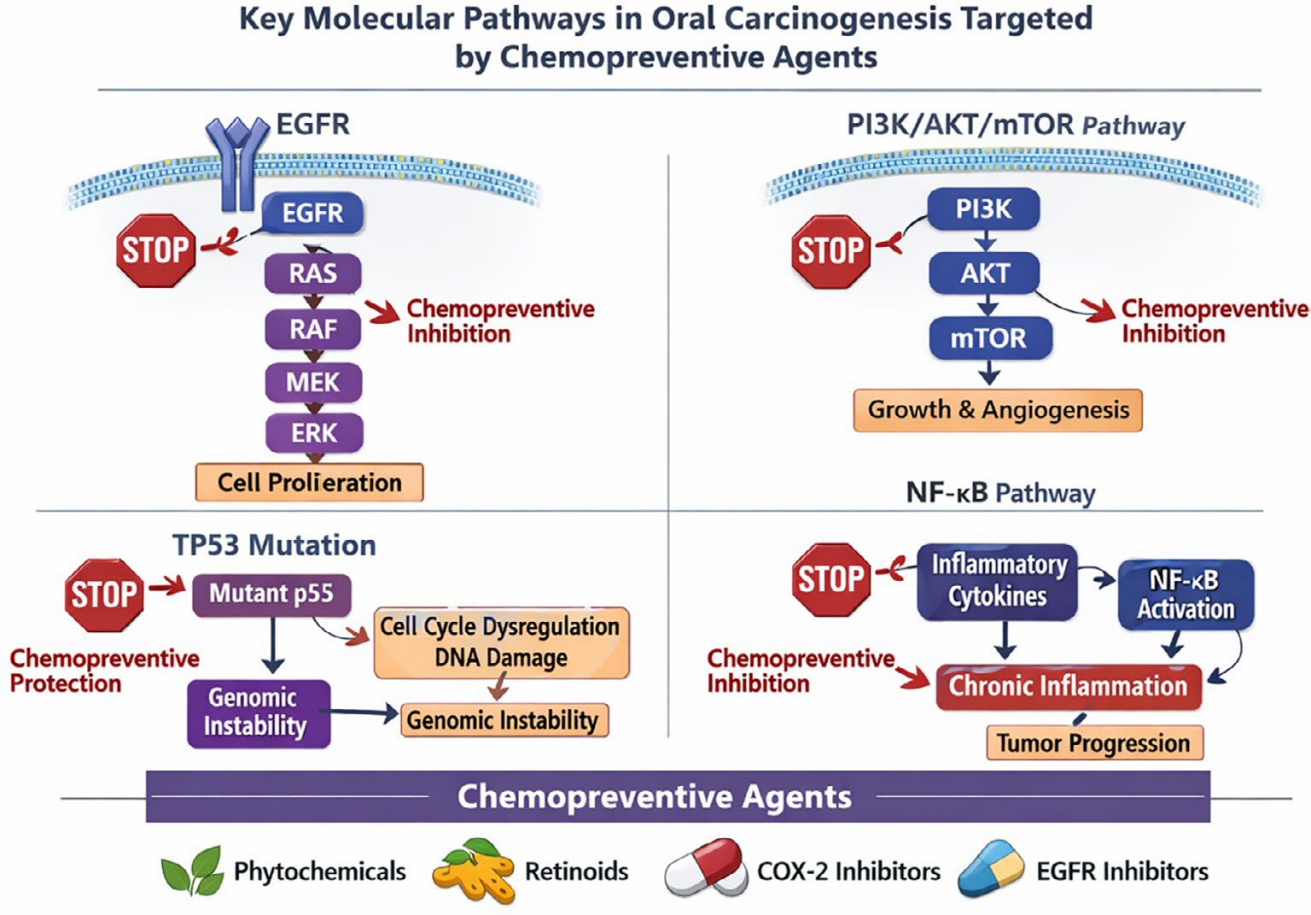
Major molecular pathways involved in oral carcinogenesis and potential targets of chemopreventive agents.

Understanding these molecular pathways provides a biological rationale for the development of chemopreventive agents, many of which exert their effects by modulating oxidative stress, inflammatory signaling, and key oncogenic pathways involved in oral carcinogenesis (Figure 1).

## 3 Oral Carcinogenesis

**FIGURE 2:**
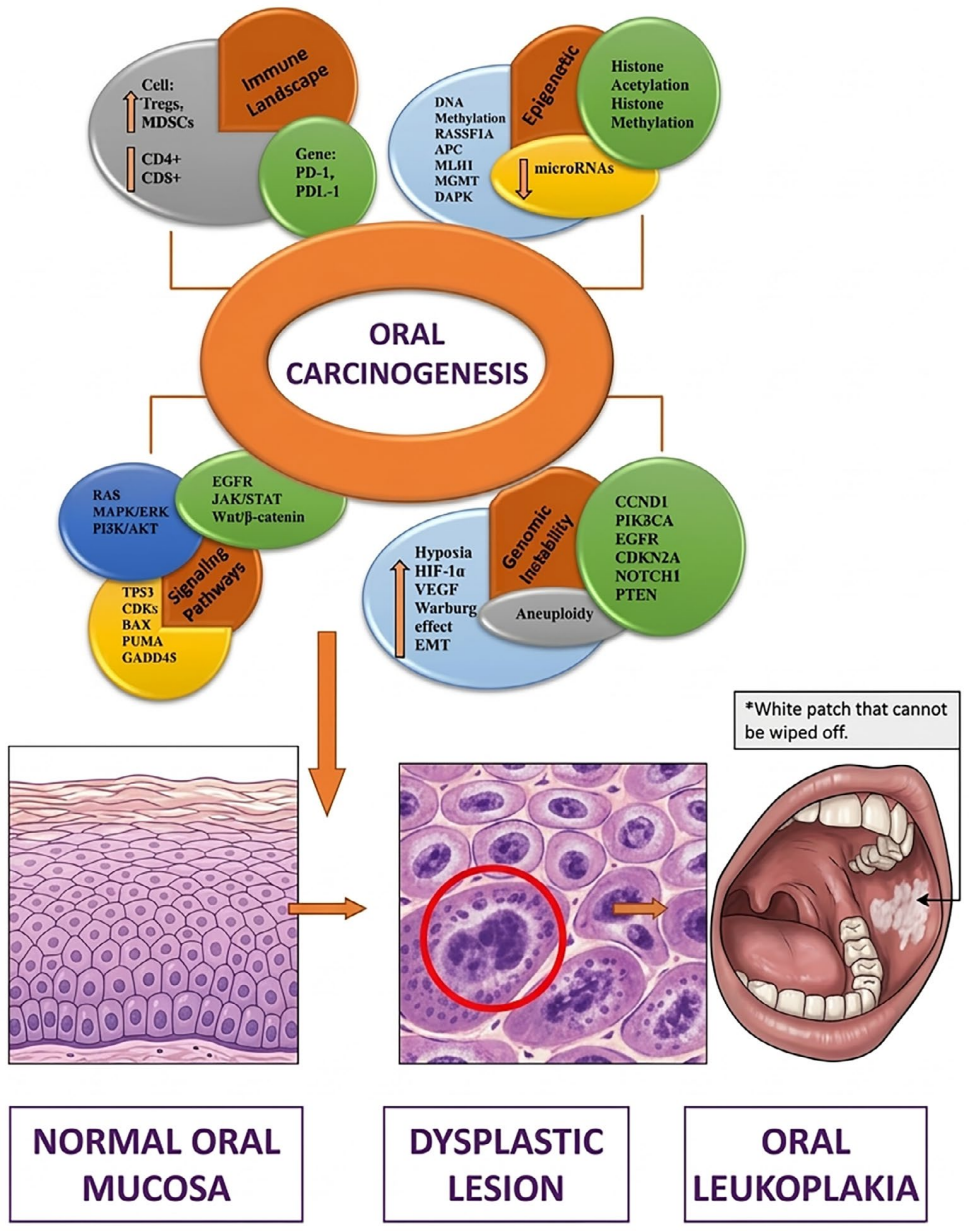
Reveals the molecular pathways involved in oral carcinogenesis.

Oral carcinogenesis is associated with a highly complex multistep process including several genetic, cytogenetic, and epigenetic modifications, which include a wide range of aberrations such as chromosomal rearrangements, mutations, and methylation. On the other hand, lifestyle factors (tobacco/areca nut/cigarettes/alcohol), which often vary with ethnic groups or geographical regions, in addition to malnutrition, hematinic and systemic sclerosis, and sexually transmitted infection of human papillomavirus (type 16), represent the primary factors related to OPMDs and OSCC [14, 15] (Figure 2).

The tumor microenvironment (TME) consists of diverse cellular and acellular components such as immune cells, cytokines, stromal cells, extracellular matrix, and the oral microbiome; the tumor microenvironment contributes to OSCC escape from immune surveillance and cytotoxic elimination [16]. This microenvironment is a complex system that includes several cell types, such as adaptive immune cells (e.g., CD4+ cells, CD8+ cells, and regulatory T cells) and myeloid immune cells (e.g., macrophages, neutrophils, monocytes, mast cells, and myeloid‐derived suppressor cells) [17] and cancer‐associated fibroblasts (CAFs) exhibit activated immunomodulatory functions, contributing to the remodeling of the extracellular matrix, immune evasion, and tumor metastasis. In addition, blood vessel endothelial cells provide plasticity, regulating the passage of fluids, oxygen, and cells. Numerous other markers and alterations in the tumor immune microenvironment (TIME) have been reported to favor the malignant transformation of OPMDs and, in some cases, the metastasis of OSCCs; these include: PD‐1, PDL‐1, TNFα [18].

Myeloid‐derived suppressor cells (MDSCs), are another critical immunosuppressive population within the OSCC tumor microenviroment. MDSCs inhibt T cell activation, promote the expansion of regulatory T cell (Tregs), and suppress anti‐tumor immune responses, production of arginase, nitric oxide, and reactive oxygen species (ROS), which impair T cell function and proliferation. Furthermore, the presence of MDSCs has been associated with increased tumor ability to invasion and resistance to conventional therapies in OSCC patients [19]. Immunosuppression is a key feature of the OSCC TME, largely mediated by (Tregs). Tregs suppress anti‐tumor immune responses by impede the activity of natural killer (NK) cells, effector T cells and through the secretion of cytokines such as interleukin‐10 (IL‐10) and transforming growth factor‐beta (TGF‐β). Increase levels of Tregs in OSCC have been correlated with reduced patient survival rates and poor prognosis [20].

Tumor‐associated macrophages (TAMs) contribute to an environment that facilitates tumor immune evasion and supports angiogenesis. The oral microbiome also plays a pivotal role in OSCC pathogenesis: dysbiosis, or imbalances in the microbiota, can induce chronic inflammation, which in turn activates carcinogenesis through the production of pro‐inflammatory cytokines, reactive oxygen species (ROS), growth factors that support tumor growth, angiogenesis, and metastasis [21]. Furthermore, these alterations affect the dysregulation of expression and function of oncogenes and tumor suppressor genes such as P53 and MCM complex proteins, as well as mitochondrial mutations [14]. For instance, loss of heterozygosity (LOH) at chromosomal regions (9p21or 3p14) in oral leukoplakia has been shown to play a significant role in the malignant progression of OPMDs. These chromosomal loci harbor tumor‐suppressor genes, including tumor protein 53 (TP53) and cyclin‐dependent kinase inhibitor 2A (CDKN2A). The efficacy of chemopreventive agents, 9p21 sustained histologic response to chemo‐preventive process using a regimen of 13‐cis‐retinoic acid and α‐tocopherol, and interferon alfa. Furthermore, genomic instability, arising from aneuploidy and accumulated mutations, contributes to tumor heterogeneity and resistance to therapy. The carcinogenesis of OSCC is also driven by key cellular alterations, including uncontrolled proliferation and evasion of apoptosis [22].

Signaling pathways such as PI3K/AKT pathways and the epidermal growth factor receptor (EGFR) are also often dysregulated, promoting OSCC cancer cell growth and survival. Angiogenesis, mediated by vascular endothelial growth factor (VEGF), facilitates metastatic spread and tumor nutrition [23, 24].

## 4 Concepts in Chemoprevention

Cancer chemoprevention, first defined by Sporn in 1976, refers to the use of natural, synthetic, or biologic chemical agents and administered either topically or systemically to reverse or prevent carcinogenic progression. Chemoprevention is broadly classified into primary, secondary, and tertiary approaches. Primary chemoprevention targets healthy individuals at high risk of developing cancer. Secondary chemoprevention is directed to individuals diagnosed with OPMDs. Tertiary chemoprevention aims to prevent recurrence or development of second primary tumors in patients who have already diagnosed and treated for cancer [25].

According to the US National Cancer Institute (NCI), most anti‐cancer medications are obtained from natural products such as classic anticancer therapies, as vincristine, vinblastine, and paclitaxel are of plant origin. Natural products are eco‐friendly, safer, low‐cost, and less toxic compared with traditional chemotherapeutic treatments and should be selective in their function and operate on cancer cells without injuring normal cells [12]. Table 1 detailed the clinical trial of chemoprevention for patients with OPMDs and OSCC.

**TABLE 1:** Clinical trial of chemoprevention for patients with OPMDs and OSCC.

Study author, year	Study design	Control/placebo	Intervention	*N*	Endpoint & results
Saran et al. (2018) [26]	RCT	Patients with Oral submucous fibrosis were divided randomly into two groups Group A (lycopene) and Group B (curcumin). Patient groups were assessed in terms of mouth opening and burning sensation.	30 pt. receive (lycopene: 4 mg, zinc: 7.5 mg, selenium: 35 mg), orally, 30 pt., treated with curcumin tablets: 300 mg and *piper nigrum* : 5 mg, period of 3 months	60	Lycopene showed better results than curcumin in improving mouth opening; both the drugs were equally effective in decreasing burning sensation in OSMF patients
Uzawa et al. (2019) [27]	Vitro + vivo study	OSCC cell lines	Cetuximab and resveratrol	—	The present findings indicate that uPAR expression plays a critical role in acquired cetuximab resistance of OSCC and that combination therapy with resveratrol may provide an attractive means for treating these patients
Islam M et al. (2023) [28]	Cross‐sectional study	One group intervention	Vitamin A, vitamin C &vitamin E were given in combination on patients using following dosage: Vitamin A (50,000) I.U., one capsule 4 days in a week for six consecutive months. Vitamin E,400 I.U., one capsule twice daily for six consecutive months. Vitamin C,500 mg, one tablet twice daily for six consecutive months	43	Study indicates potential benefits of Vitamin A, Vitamin C, and Vitamin E in mitigating oral leukoplakia symptoms, such as reduced lesion size and color improvement, with fewer adverse reactions observed
Al‐Afifi et al. (2019) [29]	RCT	Group I was provided with normal reverse osmosis (RO) water ad libitum and served as the control group. These rats were kept untreated Group II received only 4NQO‐supplemented water ad libitum and served as the induced cancer group. Group III received 4NQO‐supplemented water ad libitum and was given a vehicle (10% DMSO) orally. Groups IV, V, and VI were orally given DC extract at different concentrations 100, 500, and 1000 mg/kg respectively (for 10 consecutive weeks), starting 1 week before exposure to the carcinogen (4NQO) until 1 week after the carcinogen exposure was stopped parallel with 4NQO‐supplemented water ad libitum which was administered for eight consecutive weeks.	*Dracaena cinnabari* extract at 100, 500, and 1000 mg/kg	Vivo study involves administration of 4NQO solution for 8 weeks alone (cancer induction) or with *Dracaena cinnabari* (DC) extract at 100, 500, and 1000 mg/kg	The incidence of OSCC decreased
Swikanth Shah et al. (2021) [22]	Triple blind, pilot randomized controlled trial	—	0.1% curcumin (freshly prepared using nanoparticles) 0.15% benzydamine mouthwash 10 mL; thrice daily 6 weeks	74	Onset of oral mucositis OM 50% lower in curcumin group, Not prevent completely onset and reduce severity but delays the onset of OM

## 5 Methods

A review study based on collecting published articles that are indexed in Scopus and PubMed. All articles have been collected and reviewed by authors to ensure they're compatible with the study topic.

### 5.1 Search Strategy

A comprehensive literature search was conducted using Google Scholar, PubMed, and Scopus to identify relevant studies. Only English articles were included and assessed according to titles, abstracts, and full texts, covering the period from January 2000 to February 2025.

The search strategy employed Boolean combinations of the following terms: “oral squamous cell carcinoma”, “OSCC”, oral potentially malignant disorders, “carcinogenesis”, “chemoprevention approaches”, “treatment resistance”, “molecular targets”, “immune evasion”, and “risk factors”.

The aim of this review is to provide insight into the evolving role of chemoprevention, highlighting its potential role to progression the malignancy of OPMD and OSCC toward more preventive and controllable outcomes.

### 5.2 Eligibility Criteria

Studies were included if they met the following criteria: (a) full‐text availability publication; (b) publication in English; and (c) directly relevance to the oral cancer pathogenesis, therapeutic targets, or mechanisms of resistance associated with oral squamous cell carcinoma.

Studies were excluded if they focused on cancers other than OSCC, or were non‐original articles, including letters, editorials, and commentaries.

### 5.3 Literature Selection and Synthesis

Key information was extracted, including (i) type of chemoprevention or pathway discussed; (ii) study context (clinical, experimental, or theoretical); (iii) relation to OSCC; and oral potentially malignant disorders (iv) emerging therapeutic implications. The studies were organized into the following major categories: The role of chemoprevention in OSCC and OPMDs management; The role of the tumor microenvironment and immune evasion; Resistance mechanisms to conventional and targeted therapies; Promising molecular and immune targets and ongoing experimental therapies.

## 6 Results and Discussion

The most common chemopreventive agents in OPMDs and oral carcinogenesis are summarized in (Table 2) and they are as follows:

**TABLE 2:** Chemopreventive agents with their sources and possible mechanism of action (MoA).

Chemopreventive agents	Source (s)	Possible MoA
**Beta‐carotene**	Carrot and pepper, red peppers, sweet potatoes, pumpkin, apricots, mango, peaches, spinach, kale and watermelon	Reduce the expression of cyclin A allowing cycle Arrest, Inducing apoptosis by downregulate the activity of the anti‐apoptotic BCL‐2 and upregulate the expression of p53 A potent antioxidant that promote the activity of tumor necrosis factor‐α (TNF‐α).
Retinoid (13‐cis‐retinoic acid)	Liver, milk, eggs, fish oil, fruits, and other plants	Decreased free radicals, Suppress cell proliferation & activate programmed cell death
Lycopene	Tomatoes, cranberries, papayas, grapes, apricots, peaches, watermelon, and pink grapefruits	Administration of lycopene induced an increase in the ratio of Bax to bcl‐2, which resulted in apoptosis of cancer cells. Lycopene inhibit the epithelial‐mesenchymal transition (EMT) mechanism and the PI3K/AKT/mTOR signaling pathway
Curcumin	Dried rhizome of the *Curcuma longa* plant.	Anti‐oxidative properties (nuclear factor E2‐related factor 2, Nrf2) and inflammation by suppressing the transcription factor NF‐κB, which in turn suppresses tumorigenesis (nuclear factor kappa B, NF‐κB)
Vitamin E	Margarine and plant oils	Enhance the immune response
	Four tocopherols (alpha, beta, gamma & delta)	Destroyed cancer cell by altering cell metabolism
Resveratrol	Peanuts, mulberries, chocolate, and grape	Apoptosis, performs an anti‐inflammatory activity, inhibits the expression of RCP, indirectly down regulates the expression of EMT
Blackberries	—	Induce tumor cell apoptosis, decrease inflammatory factors, and modulate the cell's metabolism
Celecoxib	Synthetic	Celecoxib significantly delayed the growth of cancer cell and decreased tumor size. There was also reduced neo‐vascularization in the tumor sites, suggesting an anti‐angiogenic effect
Ketorolac tromethamine oral rinse	Synthetic	NSAIDs that blocks COX activity
Cetuximab	Synthetic	EGFR blocks
Erlotinib, Guggulipid	Synthetic	Blocks (EGFR)‐signal transducer and activator of transcription (STAT)‐3 signaling pathway
*Dracaena cinnabari*	Resin methanol extract	Stimulated apoptosis by up regulation of Casp3 and Bax genes and down regulation of Tp53, Cox‐2, Cyclin D1, Bcl‐2, and EGFR genes

### 6.1 Bioactive Phytochemical Compounds as Chemopreventive Agents

#### 6.1.1 Carotenoids

Carotenoids (CTDs) are a group of pigments belonging to the tetraterpenes family and are abundant in yellow to red fruits and vegetables. In human plasma, α‐carotene, β‐carotene, lycopene, β‐cryptoxanthin, lutein, and zeaxanthin collectively account for more than 95% of the total carotenoids. Nutrients like α‐, β‐, and γ‐carotene and β‐zeacarotene are easily converted into vitamin A. In contrast, non‐provitamin A includes lycopene, lutein, and zeaxanthin [30, 31].

Β‐Carotene is converted to retinol more efficiently than other provitamin A carotenoids and exhibits 100% vitamin A activity. Carotenoids have been shown to exert chemopreventive effects by reducing oncogenesis and inhibiting tumor progression at both local and generalized levels. These effects are mediated through multiple mechanisms, including regulating oxidative stress, phosphorylation, preventing ROS formation, and programmed cell death. In addition, carotenoids modulate cell proliferation, promote cell cycle arrest, induce DNA fragmentation, and alter mitochondrial membrane potential. They also influence intracellular kinase signaling and may reduce multidrug resistance (MDR) [32]. Subjects who report eating food lacking a scarcity of carotenoids have twice the risk of HNSCC carcinogenesis when compared with subjects who reported regular daily consumption [33].

#### 6.1.2 Beta‐Carotene and Vitamin A Derivates

**Beta‐carotene** is one of the major carotenoid pigmenttion found in nature. It is characterized by its red, yellowish‐orange color. Structurally, It contains of 40 carbon atoms with 11 conjugated and 2 unconjugated double bonds. It's amongst the most abundant carotenoids in the human diet and it is widely present a variety of orange, yellow and green fruits and vegetables particularly in carrot and pepper. Rich dietary sources include good dietary sources include for example, red peppers, sweet potatoes, pumpkin, apricots, mango, peaches, spinach, kale and watermelon. It exhibits potent antioxidant and anticancer properties. As a lipid‐soluble feature, carotenoids participate in many biological processes, including photosynthesis, vision process, or removing of free radicals and singlet oxygen [34]. Beta‐carotene has been shown to arrest the cell cycle by minimizing the expression of cyclin A, downregulate the activity of the anti‐apoptotic BCL‐2 and upregulate the expression of p53, leading to apoptosis of tumor cells [35, 36].

In the oral field, different studies were conducted to find the possible use of this compound against OPMDs and oral cancers. In a study performed on patients affected by leucoplakia, beta‐carotene and vitamin C were administered for 60 months. At the end of the study, the authors concluded that the administration of beta‐carotene and vitamin C had beneficial effects on the clinical improvement of the leucoplakia and malignant transformation (MT) rate lesions [37].

#### 6.1.3 Vitamin A

It is a lipid‐soluble essential vitamin that plays a critical role in morphological formation and functional maturation of epithelial cells. Retinoids and carotenoids derive from vitamin A, are synthetic analogs or natural metabolites of this vitamin. Synthetic retinoids include 13‐cis‐retinoic acid (13‐cRA), 9‐cis‐retinoic acid (9‐cRA), and 4‐hydroxy‐phenylretinamide (4‐HPR, fenretinide). Natural retinoids include all‐trans‐retinoic acid (ATRA) and etinyl palmitate (RP). Beta carotene, a precursor to vitamin A, has also been evaluated for its anti‐tumor properties. These compounds are obtained from liver, milk, eggs, fish oil, and fruits and have demonstrated notable anti‐tumor properties [38, 39, 40].

Retinoids have been shown to reduce oxidative stress by decreasing free radical formation, inducing apoptosis, and inhibiting epithelial cell proliferation. Isotretinoin (13‐cis‐retinoic acid), a vitamin A derivative, has demonstrated promising results in the management of oral leukoplakia. In a study conducted by Hong et al., isotretinoin was administered at 1–2 mg/kg per day in patients with oral leukoplakia. At the end of the study, the subjects tested showed clinical response with reductions in both lesion size and the grade of dysplasia by 54% and 67%, respectively. Despite these promising outcomes, the study highlighted notable limitations, which are the toxicity of isotretinoin and the high rate of recurrence after treatment. Consequently, a more advanced study evaluated isotretinoin in combination with vitamin E (interferon‐α and α‐tocopherol were both part of the vitamin E combination) to mitigate toxicity, prolong survival rates, and reduce the incidence of second primary tumors [41].

#### 6.1.4 Topical Formulation of Retinoids

It involves the morphogenesis, development, and differentiation of cells; hence, vitamin A deprivation led to metaplasia and de‐differentiation of epithelium. Retinoids exert most of their impact by changes in the expression of specific nuclear retinoid receptor beta messenger RNA (mRNA) expression, which arrests the progression of abnormally proliferating clones of premalignant cells of the oral cavity and skin and resets them to normal differentiation, and can prevent the initiation of second primary tumors associated with head and neck cancer cells [42].

The major method of action of retinoids is apoptosis and regulates gene expression in both normal and malignant cells by binding to RXR and RAR receptor types and e‐activator protein 1 (AP‐1). AP‐1 regulates various processes, including inflammation, oncogene function and spread [43, 44]. But the main mechanism of action to suppress cell growth and differentiation at the G1 stage is by inducing cell death or by preventing cell cycle transition. 20 Retinoids serve as chemopreventive medicines in oral potentially malignant disorders, which frequently progress to invasive squamous cell carcinoma [25].

There was a retrospective analysis examining 143 patients; the utility of oral isotretinoin rinses as chemoprophylaxis for 15 years may be beneficial in decreasing the recurrence rate of OSCC and OPMDs [45].

#### 6.1.5 Lycopene

It is the most abundant red carotene and carotenoid pigment present in tomatoes, cranberries, papayas, grapes, apricots, peaches, watermelon, and pink grapefruits which are swiftly degraded by oxidation and free radicals. It exhibits antioxidant action in biological systems [46].

Lycopene has been shown to induce apoptosis in the oral cancer cells through the regulation of BAX (a pro‐apoptotic protein) and bcl‐2 (an anti‐apoptotic protein). Specifically, lycopene increases the Bax/bcl‐2 ratio, thereby promoting apoptosis of cancer cells. Additionally, lycopene has been reported to inhibit the epithelial‐mesenchymal transition (EMT) mechanism and suppress the PI3K/AKT/mTOR signaling pathway, both of which are involved in carcinogenesis and metastasis [47, 48].

Furthermore, a study conducted by Saran et al. evaluated the therapeutic effect of lycopene and curcumin in patients with oral submucous fibrosis. 60 patients were divided randomly into two groups, group A individuals received 4 mg of lycopene and group B individuals were given 300 mg of curcumin thrice daily for 3 months. Clinical outcomes were assessed in terms of improving mouth opening and reduction in burning sensation. Lycopene showed better results than curcumin in improving mouth opening; both the drugs were equally effective in decreasing burning sensation in OSMF patients [26].

#### 6.1.6 Curcumin

Curcumin (diferuloylmethane) is derived from the dried rhizome of *Curcuma longa* (family Zingiberaceae), usually called turmeric. It consists of phytophenolic pigments, which possess antioxidant, antibacterial, antifungal, anticancer, wound healing, immunomodulatory, and anti‐inflammatory activities that act by inhibiting NF‐κB signaling, inhibiting prostaglandin synthesis, and COX‐2 expression. The most prevalent method is cell cycle inhibition and stimulation of apoptosis in cancerous cells at the G2 phase either by modulation of P53 expression or activation of the mitochondrial apoptotic pathway through enhanced BAX expression and cytochrome‐C release [49, 50].

#### 6.1.7 Polyphenolic Compounds

Polyphenols are naturally occurring compounds with a wide range of biological activities, such as scavenging of free radicals, antiviral activity, and antioxidation. Polyphenolic agents can induce oral cancer cell death and inhibition of tumor growth, invasion, and metastasis [51].

#### 6.1.8 Resveratrol

Resveratrol (3,4′,5‐trihydroxy‐trans‐stilbene) is a phytoalexin expressed by the enzymes participating in its metabolization (stilbene synthase), and naturally produced by a variety of plants, such as grapes, peanuts, chocolate and mulberries [52].

Resveratrol is a phytoestrogen, having antimicrobial, anti‐inflammatory, antioxidant and anti‐cancer activities. Despite being seen that the administration of resveratrol at daily doses of 0.5, 1.0, 2.5, or 5.0 g per day in healthy volunteers is well‐tolerated [53, 54].

In a study conducted by Kim et al., it was observed that resveratrol strongly inhibits the expression of Rab coupling protein (RCP). “RCP induces Zeb1 expression And upregulates MT1‐MMP expression to promote OSCC EMT and invasion of tumor cells”. Furthermore, resveratrol indirectly downregulates the expression of epithelial‐mesenchymal (EMT) transition, reducing the metastasis proliferation in OSCC [55].

Another study conducted by Uzawa et al. investigated alternative mechanisms underlying cetuximab‐resistance in OSCC cells. The authors demonstrated that a urokinase‐type plasminogen activator receptor (uPAR)/integrin β1/Src/FAK signal circuit converges to regulate ERK1/2 phosphorylation, thereby promoting cetuximab resistance even in the absence of EGFR‐activating mutations or EGFR overexpression. Notably, the polyphenolic phytoalexin resveratrol was shown to inhibit uPAR expression, leading to suppression of downstream ERK1/2 signaling. These results highlight the potential of resveratrol to enhance cetuximab sensitivity in OSCC both in vitro and in vivo [27].

#### 6.1.9 Blackberries

Black raspberries (BRB) and strawberries are rich sources of bioactive compounds, including flavonoids, phytochemicals, minerals, phytosterols, and vitamins. Numerous studies have demonstrated that blackberries possess significant anti‐carcinogenesis activities, including Barrett's esophagus, oral dysplasia, and colorectal cancer, particularly in in vivo studies. Similarly, dietary supplementation with strawberries has shown cancer‐preventive potential in experimental models of oral cavity, breast, lung, and anesophagus cancer [56].

BRB phytochemicals successfully reach the targeted oral tissues and modulate pro‐inflammatory and anti‐apoptotic gene expression profiles. In addition, BRB‐mediated chemoprevention has been associated with the regulation of key metabolic and signaling pathways, including glycolysis and AMP‐activated protein kinase (AMPK) pathways [57].

#### 6.1.10 Vitamin E

Epidemiological studies have found the serum vitamin E level is connected to oral cancer. The mechanism of action of Vitamin E mainly involves antioxidant activity. It is present in different common foods including margarine and plant oils. It consists of four tocopherols, such as alpha, beta, gamma, and delta. Among the four tocopherols, only α‐tocopherol is actively maintained in the human body as vitamin E. It has inhibited the development of oral cancer in an animal model [58, 59].

In a study conducted by Islam M., the efficacy and limitations of vitamin E were evaluated, demonstrating improvements in lesion size and color, with fewer adverse effects reported. However, due to the limited number of studies investigating vitamin E as a standalone agent, further research is required to better establish its efficacy as a chemopreventive strategy [28].

#### 6.1.11 Cyclooxygenase‐2 (COX‐2) Inhibitors

Cyclooxygenase (COX), which is composed of two isoforms, COX‐1 and COX‐2, is a crucial enzyme that accelerates the conversion of arachidonic acid to prostaglandins (PG). COX‐2 plays a role in the growth of epithelial malignancies, which contribute to pathological processes such as inflammation, aberrant cell apoptosis, proliferation, angiogenesis, and metastasis. The action mechanism of cyclooxygenase‐2 (COX‐2) inhibitors is believed to involve the inhibition of COX production, hence obstructing the synthesis of prostanoids, including thromboxanes (TXs) and prostaglandins (PGs) [60, 61].

### 6.2 Biological Strategies in the Management of OPMDs

#### 6.2.1 EGFR Inhibition

Epidermal Growth Factor Receptor (EGFR) is a type I receptor tyrosine kinase (RTK) located on the plasma membrane. It is expressed in numerous tissues and plays a critical function in a number of biological cellular processes important for inflammation, survival, migration, matrix homeostasis, and cell proliferation [62, 63].

#### 6.2.2 TP53/p63

TP53 is a tumor suppressor protein found on chromosome 17p13. p63 gene, a member of the p53 family, is placed on chromosome 3q27‐28, It plays a significant role in the pathogenesis of numerous human malignancies through its involvement with altered genes. There are two primary isoforms of p63: TAp63, which significantly contributes to the preservation of basal cells and prevents premature epidermal aging, and ΔNp63, which governs basal cell proliferation and initiates differentiation processes [64].

The prevalence of oral squamous cell carcinoma and tumor proliferation diminished with the administration of *Dracaena cinnabari* (DC) [29].

#### 6.2.3 Notch‐1

The Notch pathway is a prominent kind of direct cell–cell communication and plays a crucial role in proliferation, stem cell maintenance, apoptosis, and differentiation of keratinocytes [65].

Liao, S et al. proposed that curcumin has anticancer action through the inactivation of Notch‐1 and down‐regulation of NF‐κB signaling pathways, leading to the induction of apoptosis and the suppression of cell growth and invasion [66].

#### 6.2.4 Chemopreventive Agents Packed on Nanoparticles

Nanoparticles have been extensively investigated in recent years as advanced drug delivery systems to enhance the efficacy of chemopreventive agents. These nanoformulations improve bioavailability, enable sustained drug release, enhance solubility particularly for lipophilic compounds, meanwhile reduce adverse effects and potent toxicity. Conventional drugs have poor bioavailability, low solubility, short half‐life, poor stability, reduced permeability, and increased toxicity, all of which comprise their clinical effectiveness. In contrast, nanoforms (typically ranging 1–100 nm) has better bioavailability, sustained drug release, improved drug profile and in lipophilic compounds, and is a boon for drug delivery system in nanotechnology. Various nanocarrier systems have been developed, including polymeric nanoparticles, micelles, nanogels, nanofibers, and solid lipid nanoparticles [67, 68].

Numerous studies have studied the efficacy of chemopreventive agents delivered via novel drug delivery systems. In a pilot randomized controlled trial conducted by Swikanth Shah et al., the safety and effectiveness of 0.1% curcumin mouthwash (using nanoparticles) were compared with 0.15% benzydamine mouthwash in 74 head and neck cancer patients undergoing radiotherapy who developed oral mucositis (OM). The results demonstrated that 0.1% curcumin mouthwash significantly delayed the incidence of OM, highlighting the potential of nanoparticle‐based delivery systems to enhance the therapeutic effects of chemopreventive agents [69].

#### 6.2.5 Photodynamic Therapy (PDT)

Photodynamic therapy is another chemopreventive, a non‐invasive cancer treatment strategy against oral cancer and precancerous oral lesions. This therapy is based on the clinical simplicity of photosensitizer (PS) drug application followed by a specific range of light. During PDT strategy, cytotoxic reactive oxygen species (ROS) are produced by porfimer sodium (PS) excitation under specific wavelength light with oxygen (O2) supply to kill cancer cells, damaging the cancer's vascular tissue, and stimulating the immune system against the tumor [70, 71].

Among the critical parameters for optimizing photodynamic therapy (PDT), the choice of photosensitizer (PS) and tissue oxygen concentration are considered principal factors, as they enhance therapeutic efficacy while reducing side effects and improving bioavailability. Common photosensitizers include inactivated aminolevulinic acid (ALA), methylene blue, Photofrin, and chlorine‐e6. Key treatment parameters such as laser wavelength, irradiation duration, and power density typically range from 420 to 660 nm, 60 to 1000 s, and 100 to 150 mW/cm^2^, respectively [72].

Several clinical studies have investigated the use of PDT in treating oral potentially malignant disorders (OPMDs). In one study, oral erythroleukoplakia and oral verrucous hyperplasia were treated with 20% ALA gel formulated to resist saliva dilution. ALA was applied topically, followed by diode laser irradiation once weekly. Clinical improvement of the lesions was observed after approximately three treatment sessions [73].

In another study, patients with oral leukoplakia (OL) and oral lichen planus (OLP) affecting various sites, including the buccal mucosa, tongue, and gingiva, were treated with 98% topical 5‐aminolevulinic acid (5‐ALA) and irradiated with a 420 nm light‐emitting diode (LED) over multiple sessions. The results demonstrated that PDT is a feasible and effective alternative to conventional therapies for these OPMDs [74, 75].

## 7 Discussion

OSCC of the oral cavity has long been recognised as an interesting candidate for chemoprevention medicines. Because of the poor outcomes associated with the lesion, the presence of identifiable OPMDs, and the failure of local preventive therapies, such as surgery, many researchers have hoped to find effective local and systemic medications as chemopreventive compounds to hold promise as a way of diminishing the rate of morbidity and mortality associated with this disease. Many challenges are involved in the design and development of natural chemopreventive agents. These may be related to the long‐term use of chemopreventive, less side specificity and low serum bioavailability. These may be attributed to lack of rapid function after administration by any route for primary prevention without any unexpected toxicity. Several medicines, such as vitamins A, Lycopene, celecoxib, and curcumin, have been examined for potential HNSCC preventive properties via clinical trials on OPMDs [76].

Resveratrol, which has demonstrated strong anticancer, antimetastatic, and chemopreventive properties against OSCC, represents a promising candidate for the treatment of oral potentially malignant disorders (OPMDs) and OSCC [77]. Additionally, preclinical studies using blackberry‐derived polyphenols have highlighted beneficial effects on cellular metabolism, including modulation of insulin resistance and prevention of weight gain, further supporting the potential of natural compounds in chemoprevention [78].

Vitamin E has been reported to possess the ability to induce regression of epidermoid carcinomas of hamster buccal pouch when injected with a minimum essential medium [79].

The chemopreventive drug analyses, such as the Ketorolac Tromethamine Oral Rinse Cancer Prevention Trial for oral leukoplakia, found out a new plan for recognition of novel chemoprevention biomarker “to” develop new strategies for identifying novel chemoprevention biomarkers. A combinatorial method is employed for enhancement of bioavailability to acquire the synergistic effect of chemopreventive medicines. For example, isotretinoin in combination with vitamin E (interferon‐α and α‐tocopherol) as antioxidants, these dosage forms are more useful in comparison to single uses. The combinatorial approach may reduce isotretinoin toxicity and prolong survival rates. This also helps in reducing the development of a second primary tumor, exhibiting synergistic results, and providing better biological and histological outcomes [28, 41]. Newly designed chemoprevention dosages are targeted to the specific receptors such as tumor necrosis factor‐α (TNF‐α), transcription factor NF‐κB, EGFR‐signal transducer, and transcription (STAT)‐3 signaling pathway [80].

The receptors are responsible for inhibiting cell growth and inducing apoptosis, killing cancerous cells. Capsules of chemoprevention agents nanomaterials were effective in preventing OM after treating radiotherapy and chemotherapy, accelerate wound healing and can be an acceptable alternative for the current palliative and local treatments [79, 81, 82].

Hopefully, the evolution in the field of chemopreventive agents will elicit more favorable findings in the near future. It will perhaps minimise the danger of malignant transformation.

Although several chemopreventive agents have shown promising biological and clinical effects, the current evidence remains insufficient to establish standardized treatment recommendations. Future well‐designed randomized clinical trials are required to determine the efficacy and safety of these agents in preventing malignant transformation of OPMDs.

## 8 Limitations

There are a variety of problems in the creation of natural chemopreventive drugs.

- “Several of these do not exhibit the requisite instant effects.”
- “It can be challenging to calculate the timeline needed for a chemopreventive agent in order to produce an acceptable result.”
- Unanticipated toxicity when chemopreventive medicines are employed in long‐term administrations, and
- Failure of many chemopreventive studies has made researchers establish techniques to better arrange studies and evaluate expected toxicity.

## 9 Clinical Relevance

### 9.1 Scientific Rationale

OSCC & OPMDs have a high rate of morbidity and mortality. Though the necessity for less hazardous, more effective management techniques will improve the patients' lifestyle. The therapeutic potential of chemopreventive medicines as adjunct or alternative therapies in the management of oral cancer.

## 10 Pragmatic Ramifications

Findings could be exploited to guide protocols for OPMDs management, ultimately leading to the formation of standard practice incorporating chemoprevention into cancer management.

## 11 Conclusion

While multiple chemopreventive agents demonstrate biological plausibility and short‐term clinical benefits, current evidence remains insufficient to support routine clinical implementation. Well‐designed, long‐term randomized trials with malignant transformation as a primary endpoint are urgently required.

## Author Contributions

**Lana Sayal:** conceptualizing, writing the main draft, collecting data. **Omar Hamadah:** critical review. **Eyad Chatty:** supervision. **Anas Abdo:** writing the main draft. **Sana Aghbari:** collecting data. **Ali Munasser:** collecting data. **Amirah Alnour:** critical review of the manuscript.

## Funding

The authors have nothing to report.

## Conflicts of Interest

The authors declare no conflicts of interest.

## Data Availability

The data that support the findings of this study are available on request from the corresponding author. The data are not publicly available due to privacy or ethical restrictions.

## References

[1] T. S. Abbas , D. Mahdi , M. S. Ali , and A. A. Al‐fahham , “Etiology, Epidemiology and Pathophysiology of Oral Squamous Cell Carcinoma,” in A Review Article (Universal Publication Index e‐Library, 2024), 179–184.

[2] C.‐W. Chou , C. R. Lin , Y. T. Chung , and C. S. Tang , “Epidemiology of Oral Cancer in Taiwan: A Population‐Based Cancer Registry Study,” Cancers 15, no. 7 (2023): 2175. 37046836 10.3390/cancers15072175 PMC10092957

[3] O. Kujan , M. Agag , M. Smaga , et al., “PD‐1/PD‐L1, Treg‐Related Proteins, and Tumour‐Infiltrating Lymphocytes Are Associated With the Development of Oral Squamous Cell Carcinoma,” Pathology 54, no. 4 (2022): 409–416. 34872754 10.1016/j.pathol.2021.09.013

[4] J. K. L. See , X. Liu , F. Canfora , et al., “The Role of Vitamins in Oral Potentially Malignant Disorders and Oral Cancer: A Systematic Review,” Journal of Personalized Medicine 13, no. 10 (2023): 1520. 37888131 10.3390/jpm13101520 PMC10608573

[5] Z. S. Zumsteg , M. Luu , P. S. Rosenberg , et al., “Global Epidemiologic Patterns of Oropharyngeal Cancer Incidence Trends,” JNCI Journal of the National Cancer Institute 115, no. 12 (2023): 1544–1554. 37603716 10.1093/jnci/djad169 PMC10699798

[6] W. W. Y. Su , C. W. Su , D. C. Chang , et al., “Impact of Varying Anatomic Sites on Advanced Stage and Survival of Oral Cancer: 9‐Year Prospective Cohort of 27 717 Cases,” Head & Neck 41, no. 5 (2019): 1475–1483. 30652378 10.1002/hed.25579

[7] J. K. Noh , S. R. Woo , M. Kong , et al., “Gene Signature Predicting Recurrence in Oral Squamous Cell Carcinoma Is Characterized by Increased Oxidative Phosphorylation,” Molecular Oncology 17, no. 1 (2023): 134–149. 36271693 10.1002/1878-0261.13328 PMC9812830

[8] L. A. Pimenta‐Barros , P. Ramos‐García , M. Á. González‐Moles , J. M. Aguirre‐Urizar , and S. Warnakulasuriya , “Malignant Transformation of Oral Leukoplakia: Systematic Review and Comprehensive Meta‐Analysis,” Oral Diseases 31, no. 1 (2025): 69–80. 39314164 10.1111/odi.15140 PMC11808172

[9] C. Zhang , B. Li , X. Zeng , X. S. Hu , and H. Hua , “The Global Prevalence of Oral Leukoplakia: A Systematic Review and Meta‐Analysis From 1996 to 2022,” BMC Oral Health 23, no. 1 (2023): 645. 37670255 10.1186/s12903-023-03342-y PMC10481497

[10] R. Zhang , T. Gao , and D. Wang , “Photodynamic Therapy (PDT) for Oral Leukoplakia: A Systematic Review and Meta‐Analysis of Single‐Arm Studies Examining Efficacy and Subgroup Analyses,” BMC Oral Health 23, no. 1 (2023): 568. 37574560 10.1186/s12903-023-03294-3 PMC10424357

[11] R. Ellonen , A. Suominen , J. Kelppe , J. Willberg , J. Rautava , and H. Laine , “Histopathological Findings of Oral Epithelial Dysplasias and Their Relation to Malignant Transformation,” Cancer Treatment and Research Communications 34 (2023): 100664. 36481601 10.1016/j.ctarc.2022.100664

[12] A. Cardona‐Mendoza , G. Olivares‐Niño , D. Díaz‐Báez , G. I. Lafaurie , and S. J. Perdomo , “Chemopreventive and Anti‐Tumor Potential of Natural Products in Oral Cancer,” Nutrition and Cancer 74, no. 3 (2022): 779–795. 34100309 10.1080/01635581.2021.1931698

[13] M. Swetha , M. Shankar , C. K. Keerthana , T. P. Rayginia , and R. J. Anto , “Cancer Chemoprevention: A Strategic Approach Using Phytochemicals,” Frontiers in Pharmacology 12 (2022): 809308. 35095521 10.3389/fphar.2021.809308 PMC8793885

[14] L. Lorini , C. Bescós Atín , S. Thavaraj , et al., “Overview of Oral Potentially Malignant Disorders: From Risk Factors to Specific Therapies,” Cancers 13, no. 15 (2021): 3696. 34359597 10.3390/cancers13153696 PMC8345150

[15] L. G. D. S. Cabral , I. M. Martins , E. P. de Abreu Paulo , K. T. Pomini , J.‐L. Poyet , and D. A. Maria , “Molecular Mechanisms in the Carcinogenesis of Oral Squamous Cell Carcinoma: A Literature Review,” Biomolecules 15, no. 5 (2025): 621. 40427514 10.3390/biom15050621 PMC12109257

[16] M. Boxberg , L. Leising , K. Steiger , et al., “Composition and Clinical Impact of the Immunologic Tumor Microenvironment in Oral Squamous Cell Carcinoma,” Journal of Immunology 202, no. 1 (2019): 278–291. 10.4049/jimmunol.1800242 30530592

[17] C. M. Neophytou , C. Pierides , M. I. Christodoulou , P. Costeas , T. C. Kyriakou , and P. Papageorgis , “The Role of Tumor‐Associated Myeloid Cells in Modulating Cancer Therapy,” Frontiers in Oncology 10 (2020): 899. 32656079 10.3389/fonc.2020.00899 PMC7325995

[18] A. Ali , G. R. Molska , H. Yeo , et al., “Immune Microenvironment in Oral Potentially Malignant Disorders and Oral Cancer,” International Journal of Molecular Sciences 26, no. 14 (2025): 6650, 10.3390/ijms26146650. 40724900 PMC12294717

[19] R. Weber , V. Fleming , X. Hu , et al., “Myeloid‐Derived Suppressor Cells Hinder the Anti‐Cancer Activity of Immune Checkpoint Inhibitors,” Frontiers in Immunology 9 (2018): 1310. 29942309 10.3389/fimmu.2018.01310 PMC6004385

[20] I. Seminerio , G. Descamps , S. Dupont , et al., “Infiltration of FoxP3+ Regulatory T Cells Is a Strong and Independent Prognostic Factor in Head and Neck Squamous Cell Carcinoma,” Cancers 11, no. 2 (2019): 227. 30781400 10.3390/cancers11020227 PMC6406934

[21] Y. Chen , Y. Song , W. du , L. Gong , H. Chang , and Z. Zou , “Tumor‐Associated Macrophages: An Accomplice in Solid Tumor Progression,” Journal of Biomedical Science 26, no. 1 (2019): 78. 31629410 10.1186/s12929-019-0568-z PMC6800990

[22] P. A. Shah , C. Huang , Q. Li , et al., “NOTCH1 Signaling in Head and Neck Squamous Cell Carcinoma,” Cells 9, no. 12 (2020): 2677. 33322834 10.3390/cells9122677 PMC7764697

[23] H. S. Rehmani and N. Issaeva , “EGFR in Head and Neck Squamous Cell Carcinoma: Exploring Possibilities of Novel Drug Combinations,” Annals of Translational Medicine 8, no. 13 (2020): 813. 32793658 10.21037/atm.2020.04.07 PMC7396252

[24] D. E. Johnson , B. Burtness , C. R. Leemans , V. W. Y. Lui , J. E. Bauman , and J. R. Grandis , “Head and Neck Squamous Cell Carcinoma,” Nature Reviews. Disease Primers 6, no. 1 (2020): 92. 10.1038/s41572-020-00224-3 PMC7944998 33243986

[25] V. D. M. Palma , N. K. Laureano , L. A. Frank , P. V. Rados , and F. Visioli , “Chemoprevention in Oral Leukoplakia: Challenges and Current Landscape,” Frontiers in Oral Health 4 (2023): 1191347. 37293562 10.3389/froh.2023.1191347 PMC10244562

[26] G. Saran , D. Umapathy , N. Misra , et al., “A Comparative Study to Evaluate the Efficacy of Lycopene and Curcumin in Oral Submucous Fibrosis Patients: A Randomized Clinical Trial,” Indian Journal of Dental Research 29, no. 3 (2018): 303–312. 29900913 10.4103/ijdr.IJDR_551_16

[27] K. Uzawa , A. L. Amelio , A. Kasamatsu , et al., “Resveratrol Targets Urokinase‐Type Plasminogen Activator Receptor Expression to Overcome Cetuximab‐Resistance in Oral Squamous Cell Carcinoma,” Scientific Reports 9, no. 1 (2019): 12179. 31434965 10.1038/s41598-019-48717-w PMC6704133

[28] M. Islam , M. N. Ali , R. B. Anwar , et al., “The Role of Vitamin A, Vitamin C and Vitamin E for Chemoprevention of Oral Leukoplakia,” Update Dental College Journal 13, no. 2 (2023): 23–29.

[29] N. Al‐Afifi , A. Alabsi , F. Kaid , M. Bakri , and A. Ramanathan , “Prevention of Oral Carcinogenesis in Rats by *Dracaena cinnabari* Resin Extracts,” Clinical Oral Investigations 23, no. 5 (2019): 2287–2301. 30291495 10.1007/s00784-018-2685-6

[30] K. Starska‐Kowarska , “Dietary Carotenoids in Head and Neck Cancer‐Molecular and Clinical Implications,” Nutrients 14, no. 3 (2022): 531. 35276890 10.3390/nu14030531 PMC8838110

[31] G. Viglianisi , A. Polizzi , C. Grippaudo , S. Cocuzza , R. Leonardi , and G. Isola , “Chemopreventive and Biological Strategies in the Management of Oral Potentially Malignant and Malignant Disorders,” Bioengineering (Basel) 11, no. 1 (2024): 65. 38247942 10.3390/bioengineering11010065 PMC10813134

[32] R. C. Dos Santos , A. S. Ombredane , J. M. T. Souza , et al., “Lycopene‐Rich Extract From Red Guava ( *Psidium guajava* L.) Displays Cytotoxic Effect Against Human Breast Adenocarcinoma Cell Line MCF‐7 via an Apoptotic‐Like Pathway,” Food Research International 105 (2018): 184–196. 29433206 10.1016/j.foodres.2017.10.045

[33] M. Miranda‐Galvis , R. Loveless , L. P. Kowalski , and Y. Teng , “Impacts of Environmental Factors on Head and Neck Cancer Pathogenesis and Progression,” Cells 10, no. 2 (2021): 389. 33668576 10.3390/cells10020389 PMC7917998

[34] E. M. Yahia , P. García‐Solís , and M. E. M. Celis , Contribution of Fruits and Vegetables to Human Nutrition and Health, in Postharvest Physiology and Biochemistry of Fruits and Vegetables (Elsevier, 2019), 19–4520.

[35] Z. X. Lee , H. Guo , A. D. Looi , et al., “Carotenoids Modulate FoxO‐Induced Cell Cycle Arrest in Human Cancer Cell Lines: A Scoping Review,” Food Science & Nutrition 13, no. 4 (2025): e70100. 40161411 10.1002/fsn3.70100 PMC11953061

[36] L. Koklesova , A. Liskova , M. Samec , et al., “Carotenoids in Cancer Apoptosis—The Road From Bench to Bedside and Back,” Cancers 12, no. 9 (2020): 2425. 32859058 10.3390/cancers12092425 PMC7563597

[37] M. Lodolo , J. Valor , and A. Villa , “Randomized Controlled Trials for Oral Leukoplakia,” Oral Diseases 31 (2025): 3034–3038. 40457716 10.1111/odi.15399 PMC12803550

[38] M. Gürbüz and Ş. Aktaç , “Understanding the Role of Vitamin A and Its Precursors in the Immune System,” Nutrition Clinique et Métabolisme 36, no. 2 (2022): 89–98.

[39] A. Carazo , K. Macáková , K. Matoušová , L. K. Krčmová , M. Protti , and P. Mladěnka , “Vitamin A Update: Forms, Sources, Kinetics, Detection, Function, Deficiency, Therapeutic Use and Toxicity,” Nutrients 13, no. 5 (2021): 1703. 34069881 10.3390/nu13051703 PMC8157347

[40] N. Sujir , G. Priyanka , J. Ahmed , A. Saha , Y. Chhaparwal , and N. Shenoy , “Oral Cancer Chemoprevention: A Review,” Acta Marisiensis‐Seria Médica 69, no. 1 (2023): 17–22.

[41] K. Crooker , R. Aliani , M. Ananth , L. Arnold , S. Anant , and S. M. Thomas , “A Review of Promising Natural Chemopreventive Agents for Head and Neck Cancer,” Cancer Prevention Research 11, no. 8 (2018): 441–450. 29602908 10.1158/1940-6207.CAPR-17-0419 PMC6072563

[42] R. Wu , “Growth and Differentiation of Tracheobronchial Epithelial Cells,” in Lung Growth and Development (CRC Press, 2024), 211–242.

[43] G. Brown , “Retinoic Acid Receptor Regulation of Decision‐Making for Cell Differentiation,” Frontiers in Cell and Developmental Biology 11 (2023): 1182204. 37082619 10.3389/fcell.2023.1182204 PMC10110968

[44] R. Shi , M. A. al Noman , N. Mannowetz , et al., “From Discovery to Clinical Trial: YCT‐529, an Oral NonHormonal Male Contraceptive Targeting the Retinoic Acid Receptor Alpha,” Journal of Medicinal Chemistry 69 (2026): 1568–1605. 41524264 10.1021/acs.jmedchem.5c03051 PMC12833862

[45] A. Paichitrojjana and A. Paichitrojjana , “Oral Isotretinoin and Its Uses in Dermatology: A Review,” Drug Design, Development and Therapy 17 (2023): 2573–2591. 37649956 10.2147/DDDT.S427530 PMC10464604

[46] A. Kulawik , J. Cielecka‐Piontek , and P. Zalewski , “The Importance of Antioxidant Activity for the Health‐Promoting Effect of Lycopene,” Nutrients 15, no. 17 (2023): 3821. 37686853 10.3390/nu15173821 PMC10490373

[47] T. Brabletz , R. Kalluri , M. A. Nieto , and R. A. Weinberg , “EMT in Cancer,” Nature Reviews Cancer 18, no. 2 (2018): 128–134. 10.1038/nrc.2017.118 29326430

[48] R. Wang , X. Lu , and R. Yu , “Lycopene Inhibits Epithelial–Mesenchymal Transition and Promotes Apoptosis in Oral Cancer via PI3K/AKT/m‐TOR Signal Pathway,” Drug Design, Development and Therapy 14 (2020): 2461–2471. 32606612 10.2147/DDDT.S251614 PMC7321693

[49] S. Hamdy , G. E. Elshopakey , E. F. Risha , S. Rezk , A. I. Ateya , and F. M. Abdelhamid , “Curcumin Mitigates Gentamicin Induced‐Renal and Cardiac Toxicity via Modulation of Keap1/Nrf2, NF‐κB/iNOS and Bcl‐2/BAX Pathways,” Food and Chemical Toxicology 183 (2024): 114323. 38056816 10.1016/j.fct.2023.114323

[50] U. Abdull Rahim , M. Mustapa , N. N. S. Mohamed Shakrin , et al., “Current Evidence and Future Direction on Evaluating the Anticancer Effects of Curcumin, Gingerols, and Shogaols in Cervical Cancer: A Systematic Review,” PLoS One 19, no. 11 (2024): e0314280. 39576841 10.1371/journal.pone.0314280 PMC11584093

[51] Q.‐z. Lv , Q. Lv , J. Long , et al., “Current State of Knowledge on the Antioxidant Effects and Mechanisms of Action of Polyphenolic Compounds,” Natural Product Communications 16, no. 7 (2021): 1934578X211027745.

[52] B. Marant , J. Crouzet , A. L. Flourat , P. Jeandet , A. Aziz , and E. Courot , “Key‐Enzymes Involved in the Biosynthesis of Resveratrol‐Based Stilbenes in Vitis spp.: A Review,” Phytochemistry Reviews 24, no. 1 (2025): 461–481.

[53] I. Szymkowiak , J. Marcinkowska , M. Kucinska , M. Regulski , and M. Murias , “Resveratrol Bioavailability After Oral Administration: A Meta‐Analysis of Clinical Trial Data,” Phytotherapy Research 39, no. 1 (2025): 453–464. 39557444 10.1002/ptr.8379

[54] K. Brown , D. Theofanous , R. G. Britton , et al., “Resveratrol for the Management of Human Health: How Far Have We Come? A Systematic Review of Resveratrol Clinical Trials to Highlight Gaps and Opportunities,” International Journal of Molecular Sciences 25, no. 2 (2024): 747. 38255828 10.3390/ijms25020747 PMC10815776

[55] J. Y. Kim , K. H. Cho , B. Y. Jeong , C. G. Park , and H. Y. Lee , “Zeb1 for RCP‐Induced Oral Cancer Cell Invasion and Its Suppression by Resveratrol,” Experimental & Molecular Medicine 52, no. 7 (2020): 1152–1163. 32728068 10.1038/s12276-020-0474-1 PMC8080807

[56] N. Shi and T. Chen , “Chemopreventive Properties of Black Raspberries and Strawberries in Esophageal Cancer Review,” Antioxidants 11, no. 9 (2022): 1815. 36139889 10.3390/antiox11091815 PMC9495642

[57] T. J. Knobloch , N. M. Ryan , L. Bruschweiler‐Li , et al., “Metabolic Regulation of Glycolysis and AMP Activated Protein Kinase Pathways During Black Raspberry‐Mediated Oral Cancer Chemoprevention,” Metabolites 9, no. 7 (2019): 140. 31336728 10.3390/metabo9070140 PMC6680978

[58] A. J. Didier , J. Stiene , L. Fang , D. Watkins , L. D. Dworkin , and J. F. Creeden , “Antioxidant and Anti‐Tumor Effects of Dietary Vitamins A, C, and E,” Antioxidants 12, no. 3 (2023): 632. 36978880 10.3390/antiox12030632 PMC10045152

[59] P. Knekt , “Epidemiology of Vitamin E: Evidence for Anticancer Effects in Humans,” in Vitamin E in Health and Disease (CRC Press, 2023), 513–528.

[60] K. Jin , C. Qian , J. Lin , and B. Liu , “Cyclooxygenase‐2‐Prostaglandin E2 Pathway: A Key Player in Tumor‐Associated Immune Cells,” Frontiers in Oncology 13 (2023): 1099811. 36776289 10.3389/fonc.2023.1099811 PMC9911818

[61] A. Aliabadi , E. Khanniri , M. Mahboubi‐Rabbani , and M. Bayanati , “Dual COX‐2/15‐LOX Inhibitors: A New Avenue in the Prevention of Cancer,” European Journal of Medicinal Chemistry 261 (2023): 115866. 37862815 10.1016/j.ejmech.2023.115866

[62] N. Ebrahimi , E. Fardi , H. Ghaderi , et al., “Receptor Tyrosine Kinase Inhibitors in Cancer,” Cellular and Molecular Life Sciences 80, no. 4 (2023): 104. 36947256 10.1007/s00018-023-04729-4 PMC11073124

[63] M. Girych , W. Kulig , G. Enkavi , and I. Vattulainen , “How Neuromembrane Lipids Modulate Membrane Proteins: Insights From G‐Protein‐Coupled Receptors (GPCRs) and Receptor Tyrosine Kinases (RTKs),” Cold Spring Harbor Perspectives in Biology 15, no. 10 (2023): a041419. 37487628 10.1101/cshperspect.a041419 PMC10547395

[64] S. S. Srivastava , H. Alam , S. J. Patil , et al., “Keratin 5/14‐Mediated Cell Differentiation and Transformation Are Regulated by TAp63 and Notch‐1 in Oral Squamous Cell Carcinoma‐Derived Cells,” Oncology Reports 39, no. 5 (2018): 2393–2401. 29512781 10.3892/or.2018.6298

[65] D. Al Labban , S.‐H. Jo , P. Ostano , et al., “Notch‐Effector CSL Promotes Squamous Cell Carcinoma by Repressing Histone Demethylase KDM6B,” Journal of Clinical Investigation 128, no. 6 (2018): 2581–2599. 29757189 10.1172/JCI96915 PMC5983322

[66] Y. H. Liao , W. Y. Chou , C. W. Chang , et al., “Chemoprevention of Oral Cancer: A Review and Future Perspectives,” Head & Neck 45, no. 4 (2023): 1045–1059. 36810813 10.1002/hed.27301

[67] M. Rambod , N. Pasyar , and M. Ramzi , “The Effect of Zinc Sulfate on Prevention, Incidence, and Severity of Mucositis in Leukemia Patients Undergoing Chemotherapy,” European Journal of Oncology Nursing 33 (2018): 14–21. 29551172 10.1016/j.ejon.2018.01.007

[68] S. Dharman , G. Maragathavalli , R. Shanmugam , and K. Shanmugasundaram , “Current Perspectives of Nanotherapies in the Prevention and Treatment of Radiotherapy/Chemotherapy‐Induced Oral Mucositis in Head and Neck Cancer—A Narrative Review,” Journal of International Oral Health 15, no. 6 (2023): 491–499.

[69] S. Shah , H. Rath , G. Sharma , S. N. Senapati , and E. Mishra , “Effectiveness of Curcumin Mouthwash on Radiation‐Induced Oral Mucositis Among Head and Neck Cancer Patients: A Triple‐Blind, Pilot Randomised Controlled Trial,” Indian Journal of Dental Research 31, no. 5 (2020): 718–727. 33433509 10.4103/ijdr.IJDR_822_18

[70] N. Alvarez and A. Sevilla , “Current Advances in Photodynamic Therapy (PDT) and the Future Potential of PDT‐Combinatorial Cancer Therapies,” International Journal of Molecular Sciences 25, no. 2 (2024): 1023. 38256096 10.3390/ijms25021023 PMC10815790

[71] D. Hu , Y. Li , R. Li , et al., “Recent Advances in Reactive Oxygen Species (ROS)‐Responsive Drug Delivery Systems for Photodynamic Therapy of Cancer,” Acta Pharmaceutica Sinica B 14, no. 12 (2024): 5106–5131. 39807318 10.1016/j.apsb.2024.10.015 PMC11725102

[72] Y. Li , B. Wang , S. Zheng , and Y. He , “Photodynamic Therapy in the Treatment of Oral Leukoplakia: A Systematic Review,” Photodiagnosis and Photodynamic Therapy 25 (2019): 17–22. 30391342 10.1016/j.pdpdt.2018.10.023

[73] X. Liu , Q. Guo , Q. Wang , et al., “Evaluation of Long‐Term Efficacy of 5‐ALA‐PDT for Oral Potentially Malignant Disorders: A Cohort Study,” Photodiagnosis and Photodynamic Therapy 56 (2025): 104918.

[74] R. Nagi , A. Muthukrishnan , and N. Rakesh , “Effectiveness of Photodynamic Therapy (PDT) in the Management of Symptomatic Oral Lichen Planus‐A Systematic Review,” Journal of Oral Biology and Craniofacial Research 13, no. 2 (2023): 353–359. 36941903 10.1016/j.jobcr.2023.03.003 PMC10023948

[75] R. Choudhary , S. S. Reddy , R. Nagi , R. Nagaraju , S. P. Kunjumon , and R. Sen , “The Effect of Photodynamic Therapy on Oral‐Premalignant Lesions: A Systematic Review,” Journal of Clinical and Experimental Dentistry 14, no. 3 (2022): e285. 35317296 10.4317/jced.59348 PMC8916595

[76] T. Ahmad , I. Khan , M. M. Rizvi , M. Saalim , N. Manzoor , and A. Sultana , “An Overview of Effect of Lycopene and Curcumin in Oral Leukoplakia and Oral Submucous Fibrosis,” National Journal of Maxillofacial Surgery 12, no. 3 (2021): 316–323. 35153425 10.4103/njms.njms_324_21 PMC8820305

[77] G. Angellotti , G. di Prima , E. Belfiore , G. Campisi , and V. de Caro , “Chemopreventive and Anticancer Role of Resveratrol Against Oral Squamous Cell Carcinoma,” Pharmaceutics 15, no. 1 (2023): 275. 36678905 10.3390/pharmaceutics15010275 PMC9866019

[78] G. Shklar and J. L. Schwartz , “Effects of Vitamin E on Oral Carcinogenesis and Oral Cancer,” in Vitamin E in Health and Disease (CRC Press, 2023), 497–512.

[79] S. J. Kia , M. Basirat , H. S. Saedi , and S. A. Arab , “Effects of Nanomicelle Curcumin Capsules on Prevention and Treatment of Oral Mucosits in Patients Under Chemotherapy With or Without Head and Neck Radiotherapy: A Randomized Clinical Trial,” BMC Complementary Medicine and Therapies 21, no. 1 (2021): 232. 34521398 10.1186/s12906-021-03400-4 PMC8442420

[80] H. Sarma , T. Jahan , and H. K. Sharma , “Progress in Drug and Formulation Development for the Chemoprevention of Oral Squamous Cell Carcinoma: A Review,” Recent Patents on Drug Delivery & Formulation 13, no. 1 (2019): 16–36. 30806332 10.2174/1872211313666190222182824

[81] R. Paliwal and S. R. Paliwal , “Controlled Delivery of Chemopreventive Agents,” in Advances in Nanochemoprevention: Controlled Delivery of Phytochemical Bioactives (Springer, 2021), 29–38.

[82] A. Prabhu , A. Prabhu , V. Baliga , et al., “Transforming Wound Management: Nanomaterials and Their Clinical Impact,” Pharmaceutics 15, no. 5 (2023): 1560. 37242802 10.3390/pharmaceutics15051560 PMC10221108

